# A platform of BRET-FRET hybrid biosensors for optogenetics, chemical screening, and *in vivo* imaging

**DOI:** 10.1038/s41598-018-27174-x

**Published:** 2018-06-12

**Authors:** Naoki Komatsu, Kenta Terai, Ayako Imanishi, Yuji Kamioka, Kenta Sumiyama, Takashi Jin, Yasushi Okada, Takeharu Nagai, Michiyuki Matsuda

**Affiliations:** 1 Laboratory of Bioimaging and Cell Signaling, Graduate School of Biostudies, Kyoto University, Kyoto, Japan; 2 Department of Pathology and Biology of Diseases, Graduate School of Medicine, Kyoto University, Kyoto, Japan; 3 Laboratory for Mouse Genetic Engineering, Quantitative Biology Center, RIKEN, Suita, Japan; 4 Laboratory for Nano-Bio Probes, Quantitative Biology Center, RIKEN, Suita, Japan; 5 Laboratory for Cell Polarity Regulation, Quantitative Biology Center, RIKEN, Suita, Japan; 6 The Institute of Scientific and Industrial Research, Osaka University, Suita, Japan; 7 Present Address: Laboratory for Cell Function Dynamics, Center for Brain Science, RIKEN, Wako, Japan; 8 Present Address: Department of Molecular Genetics, Institute of Biomedical Science, Kansai Medical University, Hirakata, Japan

## Abstract

Genetically encoded biosensors based on the principle of Förster resonance energy transfer comprise two major classes: biosensors based on fluorescence resonance energy transfer (FRET) and those based on bioluminescence energy transfer (BRET). The FRET biosensors visualize signaling-molecule activity in cells or tissues with high resolution. Meanwhile, due to the low background signal, the BRET biosensors are primarily used in drug screening. Here, we report a protocol to transform intramolecular FRET biosensors to BRET-FRET hybrid biosensors called hyBRET biosensors. The hyBRET biosensors retain all properties of the prototype FRET biosensors and also work as BRET biosensors with dynamic ranges comparable to the prototype FRET biosensors. The hyBRET biosensors are compatible with optogenetics, luminescence microplate reader assays, and non-invasive whole-body imaging of xenograft and transgenic mice. This simple protocol will expand the use of FRET biosensors and enable visualization of the multiscale dynamics of cell signaling in live animals.

## Introduction

Förster resonance energy transfer is a form of energy transfer from a donor molecule to an acceptor molecule. Based on this principle, two types of genetically encoded biosensors have been developed^1–5^. Biosensors based on fluorescence resonance energy transfer (FRET) use fluorescent proteins as the donor, while those based on bioluminescence resonance energy transfer (BRET) use bioluminescent proteins as the donor. The FRET biosensors have been broadly used to visualize the intracellular activities of signaling molecules such as protein kinases and small GTPases^3,5^. However, they suffer from problems that are inherent to fluorescence imaging, including (1) background fluorescence from cellular components and chemical compounds, (2) photo-toxicity of excitation light, (3) photo-bleaching of the fluorophores, (4) incompatibility with optogenetic tools, and (5) invasive procedures for *in vivo* microscopy^1,2^. BRET biosensors are ideal tools to circumvent these problems and, in fact, have been used not only to detect protein-protein interactions within cells and tissues^6–8^, but also for drug development^9–11^. Intuitively, genetically encoded biosensors based on BRET could be designed similarly to the FRET biosensors, because the only difference between the two types of biosensors is the donor proteins. However, simple replacement of the donor fluorescent protein in the FRET biosensors with a donor bioluminescent protein does not work in many cases. Moreover, the bioluminescence-based biosensors often suffered from low intensity of light emission and short half-life of the substrate^1^. Recent development of an extremely bright luciferase, NanoLuc, may overcome this problem^12^, but currently, genetically encoded biosensors for signaling molecules are mostly based on FRET rather than BRET due to the aforementioned reasons.

Recently, Saito *et al*. developed a brighter bioluminescent protein, Nano-lantern, by fusing a fluorescent protein to Renilla luciferase (RLuc)^13^. A subsequent study revealed that various fluorescent proteins can be fused to RLuc to develop a number of color variants of Nano-lantern^14^. Inspired by this work, we aimed to develop biosensors that employ RLuc fused to a fluorescent protein as the donor of BRET biosensors. The rationale is as follows. Optimizations of fluorescent proteins suitable for FRET biosensors have been extensively performed by many research groups^15,16^. Therefore, we could expect to accelerate the development of BRET biosensors by preserving the backbone of the prototype FRET biosensors. Moreover, many of the FRET biosensors reported so far are comprised of a cyan fluorescent protein (CFP) and a yellow fluorescent protein (YFP). If we could use a CFP fused to RLuc as the donor, many FRET biosensors could be transformed to BRET biosensors. In fact, Aper *et al*. have reported that a fusion protein of NanoLuc and CFP could serve as the donor for YFP to generate a biosensor for intracellular Zinc^17^.

Here, we have developed a genetically encoded intramolecular ERK activity biosensor comprising a yellow fluorescent protein (YFP), a cyan fluorescent protein (CFP) and the bioluminescent protein RLuc8, and monitored the ERK activity by either BRET or FRET. In the BRET mode, the energy is transferred from RLuc8 to CFP, and then from CFP to YFP. Following this success, we show that many FRET biosensors could be easily transformed to BRET biosensors. As anticipated, the new FRET-BRET hybrid biosensors are compatible with optogenetic manipulations and cell-based assays with a microplate luminescent reader. Finally, we show that the pharmacodynamics of ERK activity in live mice could be monitored non-invasively, paving the way to *in vivo* application of FRET biosensors.

## Results

### Transformation of FRET biosensors to BRET biosensors

**Figure 1:**
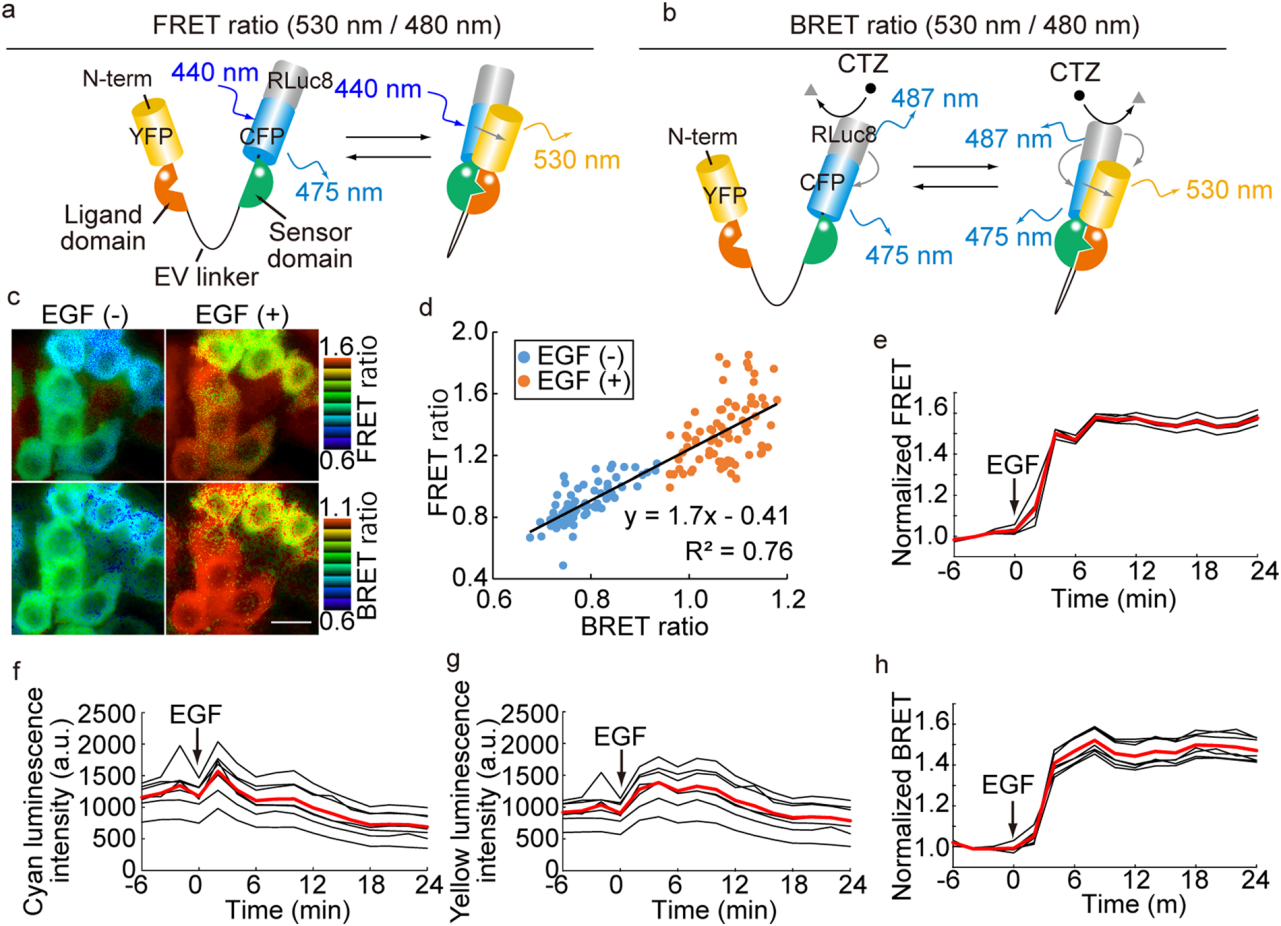
hyBRET biosensor for BRET and FRET imaging. (**a**,**b**) Mechanism of action of the hyBRET biosensor in FRET (**a**) or BRET (**b**) mode. In FRET mode, the emission intensity of YFP at 530 nm over that of CFP at 480 nm is used to calculate the FRET ratio. In BRET mode, the YFP emission intensity over the summed emission intensity of CFP and RLuc8 emission is used to calculate the BRET ratio. (**c**–**j**) HeLa cells expressing hyBRET-ERK were stimulated with 10 ng/ml EGF. (**c**) FRET (upper panels) and BRET (lower panels) ratio images are shown in the IMD mode. Bar, 20 μm. (**d**) The FRET ratio and BRET ratio were quantified for each cell and shown in the scatter plot. Data are for 79 cells in four viewfields. (**e**) Representative time-courses of FRET ratio, normalized to the average before EGF stimulation. (**f**–**h**) Representative time-courses of the cyan luminescence intensity, yellow luminescence intensity, and normalized BRET ratio. Red lines show the mean value of six cells.

To transform a FRET biosensor into a BRET biosensor, RLuc8 S257G (RLuc8), a bright RLuc mutant^14,18^, was fused to the C terminus of the CFP of EKAREV, an ERK biosensor with a long flexible EV linker^19^ (Fig. 1a,b). The resulting FRET-BRET hybrid-biosensor was named hyBRET-ERK. In the FRET mode, phosphorylation of the sensor domain of hyBRET-ERK causes intramolecular association of CFP and YFP and thereby increases the FRET efficiency (Fig. 1a). In the BRET mode, upon the addition of coelenterazine-h, the energy produced by RLuc8 is non-radiatively transferred to YFP or CFP. In the latter case, excited CFP then transfers energy to YFP (Fig. 1b). We routinely used YPet and Turquoise2-GL as the donor CFP and acceptor YFP, respectively, because of high dynamic range (Fig. S1). To prove the concept, the hyBRET-ERK biosensor was expressed in HeLa cells and imaged for both fluorescence and bioluminescence in the presence of coelenterazine-h (Fig. 1c–h). The fluorescence emission intensity at 530 nm over that at 480 nm, hereafter called the FRET ratio, was used for the evaluation of FRET. Similarly, the bioluminescence emission intensity at 530 nm over that at 480 nm, called the BRET ratio, was used to evaluate the level of BRET. The FRET ratio in each cell was linearly correlated with the BRET ratio before and after EGF stimulation (Fig. 1d). The BRET ratio was always lower than the FRET ratio because the bioluminescence from RLuc8 is also detected at 480 nm. We did not find significant difference in the range of the EGF-induced increase in the FRET ratio between the prototype FRET biosensor and the hyBRET-ERK (Fig. 1e)^19^. Although both the cyan and yellow luminescence intensities were decreased during the observation period due to the decay of coelenterazine-h, the BRET ratio was robust to this decrease in the luminescence intensity (Fig. 1f–h). Moreover, the dynamic range of the BRET ratio was almost equal to that of the FRET ratio. Thus, the simple in-frame fusion of RLuc8 to the C-terminus of CFP was demonstrated to transform the FRET biosensor to a FRET-BRET hybrid biosensor.

### Estimation of energy transfer rates

**Figure 2:**
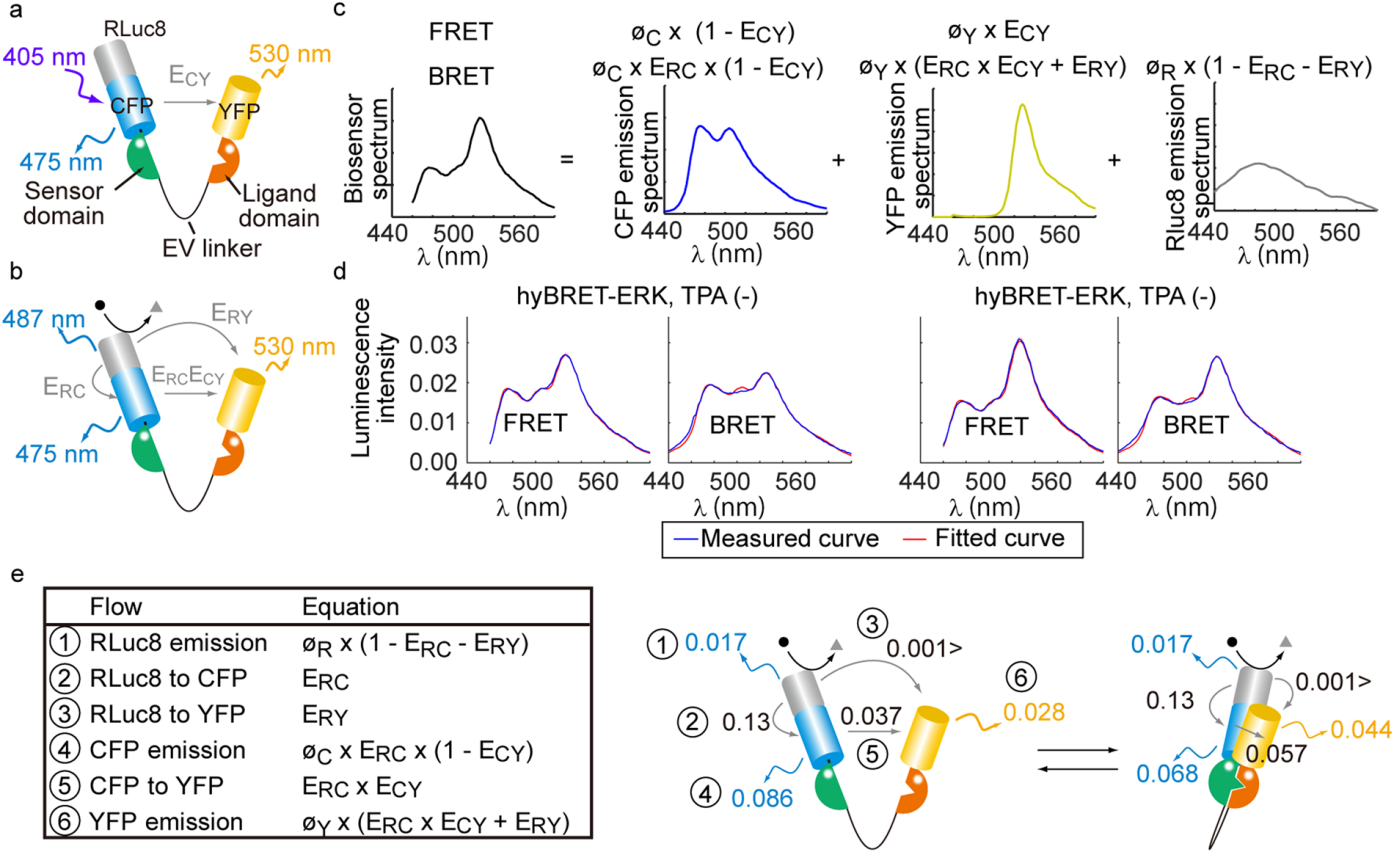
Energy transfer rates in FRET and BRET modes. (**a**–**c**) Schemes of the estimation of energy transfer rates and radiative decay rates. The fluorescent (**a**) and bioluminescent (**b**) spectra of hyBRET sensors were fitted with the spectra of CFP, YFP, and RLuc8 (**c**) by the nlinfit function of MATLAB to estimate the parameters. (**d**) Overlay of the measured (blue) and fitted (red) spectra of hyBRET-ERK before and after TPA stimulation. Envelopes with light red show the 95% confidence interval in the fitted curves. The y-axis indicates the emission intensity normalized by the area of spectrum. (**e**) Summary of the energy flow absorbed by RLuc8. Rates of energy transfer and radiative decay are shown. *E*_*RC*_, *E*_*RY*_, and *E*_*CY*_ are the energy transfer rates from RLuc8 to CFP, RLuc8 to YFP, and YFP to CFP, respectively. *ϕ*_*C*_, *ϕ*_*Y*_, and *ϕ*_*R*_ are the quantum efficiencies of CFP, YFP, and RLuc8, respectively.

YFP in the hyBRET biosensor may accept energy directly from RLuc8 or indirectly from the CFP excited by the energy transfer from RLuc8 (Fig. 2a,b). In fact, the spectral overlay between RLuc8 emission and YFP excitation is larger than that between RLuc8 emission and CFP excitation. Therefore, the contribution of each pathway was estimated by linear unmixing as described in the Materials and Methods section using the parameters reported previously (Table S1). First, as a reference, we recorded the bioluminescence spectrum of RLuc8 and fluorescence spectra of CFP and YFP expressed in HeLa cells by using a photonic multichannel analyzer (Fig. 2c). Second, HeLa cells expressing hyBRET biosensors were excited at 438 nm to record the fluorescence spectra (Fig. S2). The fractions of fluorescence from CFP and YFP were calculated by curve fitting of each reference spectrum to the fluorescence spectra of hyBRET biosensors by using the nlinfit function of MATLAB^20^. Taking the quantum efficiency of each fluorescent protein into consideration, energy transfer rates from CFP to YFP (*E*_*CY*_) were calculated. Third, HeLa cells expressing hyBRET biosensors were incubated in the presence of coelenterazine-h to record the luminescence spectra. The fractions of luminescence from RLuc8, CFP, and YPF were calculated by curve fitting, assuming that *E*_*CY*_ was constant both in the FRET and BRET modes. In this way, the energy transfer rates from RLuc8 to YFP (*E*_*RY*_) were obtained. To examine the dynamic range of hyBRET-ERK, serum-starved HeLa cells were stimulated with 12-O-Tetradecanoylphorbol 13-acetate (TPA).

Based on the FRET spectra, the energy transfer rates between CFP and YFP (*E*_*CY*_) were estimated to be 0.29 and 0.44 before and after TPA stimulation, respectively (Fig. S2 and Table S2). The BRET efficiency between RLuc8 and CFP, *E*_*RC*_, was calculated as 0.13 from the quantum efficiencies of RLuc8 and cyan Nano-lantern^14^. Assuming that *E*_*RC*_ was not changed by biosensor phosphorylation, and that *E*_*CY*_ was not changed between the FRET and BRET mode, the rates of direct energy transfer from RLuc8 to YFP (*E*_*RY*_) were estimated to be less than 0.001 irrespective of TPA stimulation (Fig. S2). Meanwhile, the CFP-mediated energy transfer rates from RLuc8 to YFP, *E*_*RC*_ × *E*_*CY*_ were 0.037 and 0.057 before and after TPA stimulation, respectively (Fig. 2e). Therefore, the CFP-mediated pathways primarily contributed to the excitation of YFP. The *E*_*CY*_ value of hyBRET-ERK-TA, in which the threonine residue in the kinase substrate domain was replaced with alanine, was 0.22, corresponding to the level of basal FRET signal of hyBRET-ERK. When energy transfer from RLuc8 to CFP was blocked by using a non-fluorescent mutant of CFP in hyBRET-ERK-W66G, *E*_*RY*_ was significantly increased (Fig. S2), although the net luminescence intensity was markedly lower than for hyBRET-ERK.

Recently, the NanoLuc-fused fluorescent protein was shown to exhibit higher luminescence intensity than the RLuc8-fused fluorescent protein^21^. Again, color variants have been generated by using various fluorescent proteins^22^, prompting us to examine whether RLuc8 could be replaced with NanoLuc. We substituted two NanoLuc-fused Turquoise proteins^21,22^ for the RLuc8-fused Turquoise protein and analyzed the energy transfer rates (Fig. S2). Both constructs, hyBRET-ERK-NanoLuc-6075 and hyBRET-ERK-NanoLuc-6088, exhibited substantial energy transfer from NanoLuc to YPet; the energy transfer rates, *E*_*NC*_, of these biosensors were 2.6-fold higher than the *E*_*RC*_. However, neither of the NanoLuc-based biosensors responded to TPA. We also constructed a biosensor named hyBRET-ERK-NanoLuc-6110, which carried NanoLuc-fused Turquoise at the N-terminus, but, again, could not observe a response to TPA (Fig. S2). Unexpectedly, these constructs carrying NanoLuc at either the N- or C-termini did not respond to TPA even in the FRET mode, implying that NanoLuc may perturb TPA-dependent phosphorylation or conformational change of the biosensor. We concluded that extensive optimization of the biosensor is needed to generate NanoLuc-based hyBRET biosensors. Lastly, we show the fluorescence profile of the FRET biosensor without RLuc8, EKAREV-4464 (Fig. S2). The FRET level is higher than that of hyBRET-ERK, but the gain after TPA stimulation was similar to that of hyBRET-ERK.

These results suggested that RLuc8, but not NanoLuc, could be fused to the other intramolecular FRET biosensors that carry CFP at the C-terminus. As expected, all FRET biosensors for PKA, S6K, JNK, Crk, and HRas, which carry CFP at the C-terminus, exhibited a stimuli-dependent increase in BRET to levels comparable to the increase in FRET (Fig. S3). Thus, when CFP is located in the C-terminus of the FRET biosensor, the in-frame fusion of RLuc8 is a versatile one-step method for transforming the FRET biosensors of serine/threonine kinase, tyrosine kinases, and small GTPases to hybrid FRET-BRET biosensors.

### Compatibility with optogenetic tools

**Figure 3:**
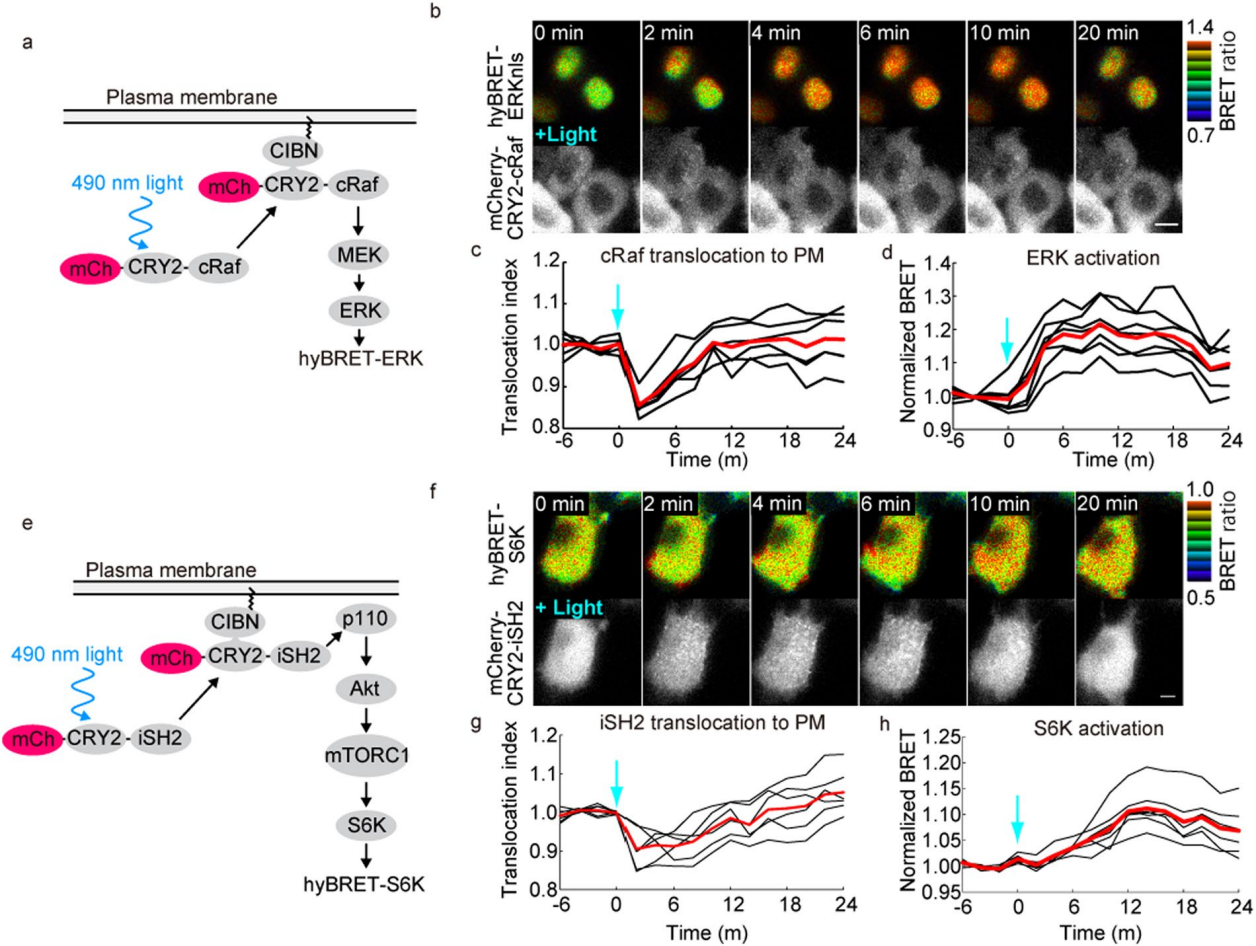
Compatibility with optogenetic tools. (**a**,**e**) Scheme of light-induced activation of ERK or S6K. HeLa cells expressing hyBRET-ERKnls and mCherry-CRY2-cRaf (**b–d**) or hyBRET-S6K and mCherry-Cry2-iSH2 (**f**–**h**) were illuminated with 490 nm light for 100 ms. The BRET image in IMD mode and mCherry fluorescence image are shown in (**b**,**f**). (**c**,**g**) The cytoplasmic intensity of mCherry was normalized to the mean value before stimulation and used as a translocation index. (**d**,**h**) The BRET ratio normalized to the mean value before stimulation was used as normalized BRET. Averages of single cells are shown with the mean value (red). Bars, 10 μm.

Many FRET biosensors are incompatible with optogenetic tools, because both optogenetic tools and FRET biosensors are often excited by blue-to-green light. To demonstrate that hyBRET biosensors could overcome this drawback, we employed an *Arabidopsis* cryptochrome 2 (CRY2) optogenetic switch^23^; in this switch, upon blue light illumination, a CRY2-fused signaling molecule binds to the plasma membrane targeted N-terminus of CIB1 (CIBN), and thereby activates signaling molecules such as Ras and phosphatidylinositol-3 kinase (PI3K)^24,25^. First, we expressed an mCherry-tagged CRY2-cRaf fusion protein and the plasma membrane-targeted CIBN in hyBRET-ERK-expressing HeLa cells (Fig. 3a). Illumination of the cells with 490 nm light induced transient translocation of mCherry-CRY2-cRaf from the cytosol to plasma membrane and ERK activation as monitored by an increase in the BRET ratio (Fig. 3b,c). Similarly, by using mCherry-CRY2-fused inter-SH2 domain of the p110 subunit of PI3K, activation of S6K was observed upon cyan light illumination (Fig. 3e–h). We also extended this approach with a CRY2-fused guanine nucleotide exchange factor for Ras and detected Ras activation at the plasma membrane (Fig. S4).

During the course of these experiments, we happened to notice that illumination of the hyBRET-ERK-expressing cells at 440 nm decreased the cyan bioluminescence and increased the BRET ratio (Fig. S5). When 490 nm light was applied, neither the cyan bioluminescence intensity nor the BRET ratio was affected. Moreover, cells expressing RLuc8 alone also exhibited a 440 nm light-dependent decrease in cyan bioluminescence intensity. Although the bioluminescence intensity was restored within 2 min, it should be kept in mind that extensive excitation at 440 nm wavelength may cause transient suppression of RLuc8.

### Live-cell analysis of ERK activity with a luminescence microplate reader

**Figure 4:**
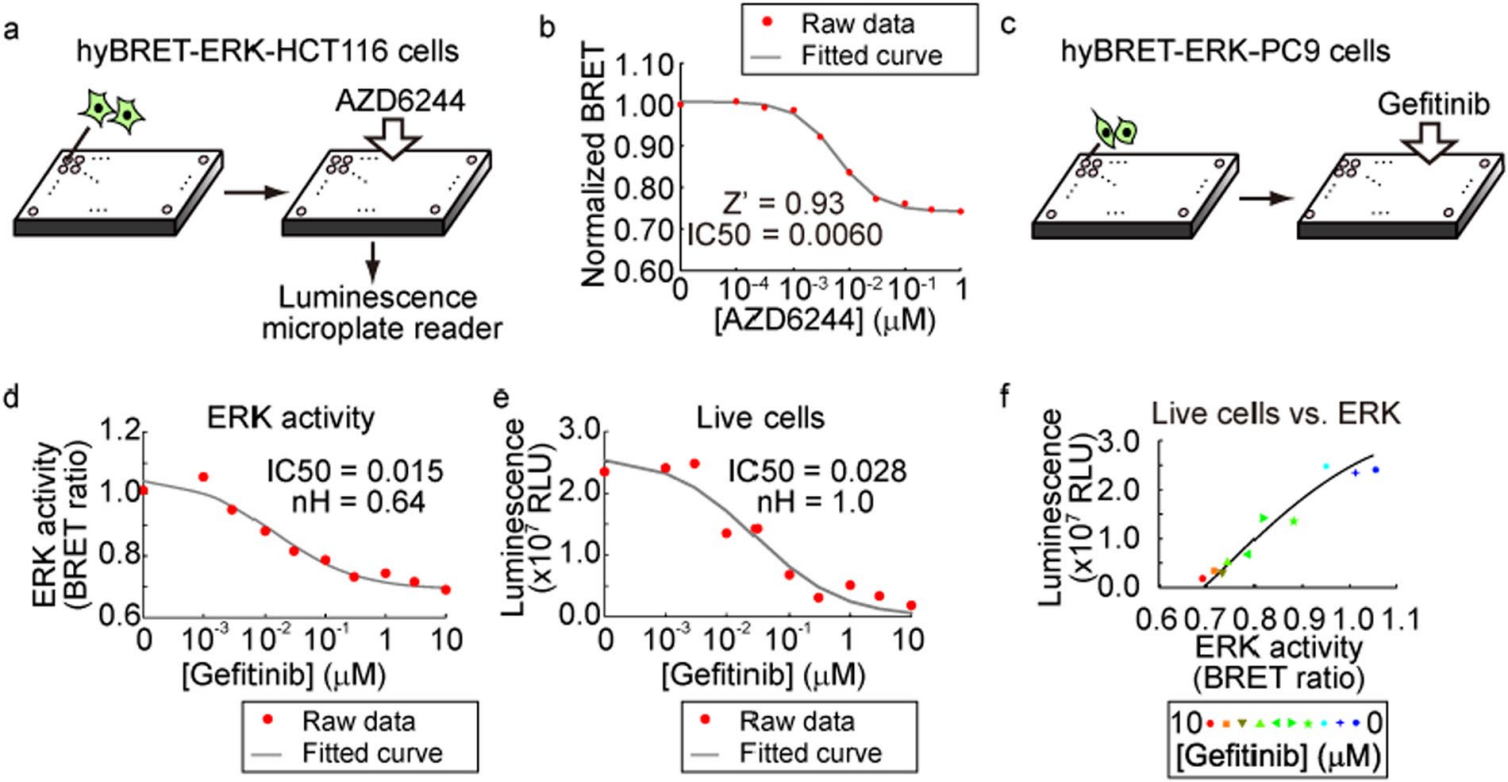
Microplate luminescent reader assay for ERK activity. (**a**) HCT116 cells expressing hyBRET-ERK were plated in 96-well plates and treated with serially diluted AZD6244, an MEK inhibitor. BRET was measured with a microplate luminescent plate reader. (**b**) Dose-dependent decrease of ERK activity by AZD6244 in HCT116 cells. Eight wells were used for each condition. The experiment was performed in triplicate. (**c**) Scheme of the multiplexed cell-based assay for ERK activity, cell viability and cell death. (**d**–**f**) PC9 cells expressing hyBRET-ERK were plated in 96-well plates and treated with serially diluted gefitinib, an EGFR inhibitor. ERK activity and live cells were quantitated by the BRET ratio (**d**) and the sum of cyan and yellow luminescence intensities (**e**), respectively. Dose-response curves of gefitinib for ERK activity (**d**) and live cells (**e**). Each dot is the average of two wells. Data were fitted by the Hill equation. (**f**) Correlation of ERK activity versus the number of live cells. For more details, see the Materials and Methods.

The application of FRET biosensors to high-throughput screening is limited in part due to the low signal-to-noise ratio in fluorescent microplate reader-based assays. Therefore, we examined whether the hyBRET biosensor could be applied for luminescence microplate reader-based screening in HCT116 colon cancer cells and PC9 lung cancer cells. HCT116 cells expressing hyBRET-ERK were incubated with serially diluted AZD6244, an MEK inhibitor, and their ERK activity was examined with a luminescence microplate reader (Fig. 4a). From the dose-response curve, the IC50 of AZD6244 was determined to be 0.0060 μM (Fig. 4b), which was comparable to the value obtained in our previous report by FRET microscopy^26^. The Z’ value was 0.93, indicating that the cell-based hyBRET assay exhibited reliability sufficient for high throughput screening.

Next, a multiplexed assay platform was established for the anti-cancer drug and EGFR inhibitor gefitinib. We expressed hyBRET-ERK in EGFR inhibitor-sensitive PC9 lung adenocarcinoma cells^27^, treated the cells with gefitinib for one day, and quantified the ERK activity by means of the BRET ratio and the living cells by the total luminescent counts (Fig. 4c–f). The IC50 values for ERK activity and cell growth were comparable: 0.015 and 0.028 μM, respectively. Moreover, the ERK activity was linearly correlated with the number of live cells, indicating that ERK activity primarily determined the sensitivity to gefitinib in PC9 cells. We also examined the sensitivity of PC9 cells to the MEK inhibitor AZD6244 (Fig. S6). The IC50 values were reliably determined for as few as 100 PC9 cells per well.

### Non-invasive *in vivo* pharmacodynamics in tumors and whole bodies

Owing to the low background of bioluminescence in tissues, the BRET-based biosensors are anticipated to be applicable for non-invasive *in vivo* imaging. However, the short half-life of coelenterazine-h has been hampering the application of RLuc8-based biosensors. To overcome the short half-life of coelenterazine-h, diacetyl coelenterazine-h has been developed^14^. We compared the *in vivo* half-lives of coelenterazine-h and diacetyl coelenterazine-h by using subcutaneously transplanted HeLa cells expressing cyan and yellow Nano-lanterns (Fig. S7). When coelenterazine-h was used as the substrate, the bioluminescence decayed rapidly close to the detection limit at 5 min after injection^28^. On the other hand, in the diacetyl coelenterazine-h-injected mice, the half-life of bioluminescence was approximately 9 min.

**Figure 5:**
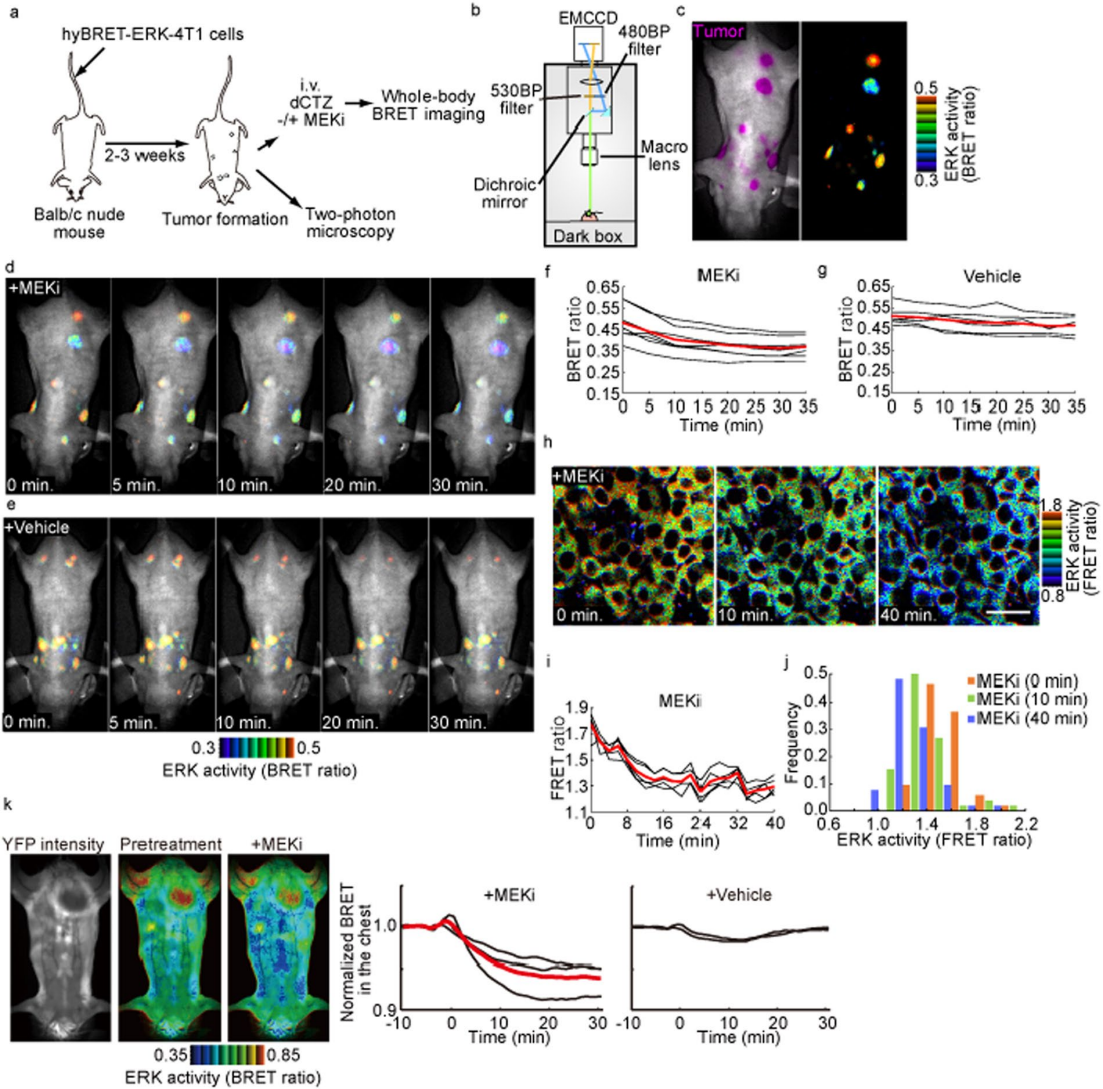
Non-invasive *in vivo* BRET imaging of ERK activity. (**a**) 4T1 mammary cancer cells expressing hyBRET-ERK were intravenously injected into Balb/c nude mice. Before image acquisition, 5 mg/kg diacetyl coelenterazine-h (dCTZ) was injected with or without the MEK inhibitor PD-0325901 (MEKi). (**b**,**c**) Anesthetized mice were subjected to bright field imaging and cyan and yellow bioluminescence imaging. Overlays of a bright-field image and cyan luminescent image (left) and BRET ratio image (right) are shown. (**d**,**e**) Time-lapse bright field and BRET ratio images. (**f**,**g**) For each tumor, average BRET ratios are plotted against time with mean values (red lines). (**h**–**j**) The lung tumor was exposed and observed under a two-photon microscope. (**h**) Shown here are representative FRET ratio images of lung tumors before (left), 10 min after (middle) and 40 min after (right) the administration of 5.0 mg/kg PD-0325901. Bar, 30 μm. (**i**) Time courses of FRET ratio for regions including 4–15 cells are shown with the mean (red line). (**j**) A histogram of ERK activity of 4T1 cells. (**k**) Transgenic mice expressing hyBRET-ERK were anesthetized and administered diacetyl coelenterazine-h by continuous intravenous infusion. At time zero, the MEK inhibitor PD-0325901 (MEKi) was injected intraperitoneally. Shown here are a representative YFP image, BRET images before and after MEK inhibitor injection, and time-lapse BRET ratios in the chest region. Black lines in the plots are data from individual mice and the red line is the average of three mice.

Encouraged by this prolonged half-life of diacetyl coelenterazine-h, we attempted to set up a non-invasive pharmacodynamics assay in mice. For this purpose, we intravenously injected 4T1 murine breast tumor cells expressing hyBRET-ERK into Balb/c nude mice (Fig. 5a). Two to three weeks after the injection, BRET signals from the implanted tumors were monitored by injecting diacetyl coelenterazine-h (Fig. 5b,c). The bioluminescence signals were primarily detected from the lung tumors. The BRET ratio ranged from 0.35 to 0.65. Upon injection of the MEK inhibitor PD-0325901, the BRET ratio decreased about 25% within 35 min, demonstrating that the effect of the MEK inhibitor could be monitored in living mice expressing the hyBRET biosensor (Fig. 5d,f). The decrease in the BRET ratio was marginal in the mice injected with the vehicle alone (Fig. 5e,g). To confirm the specificity of the change in the BRET ratio, we set up a similar experiment by using 4T1 cells expressing hyBRET-ERK-TA (Fig. S8). The BRET ratio from tumors expressing the hyBRET-ERK-TA ranged from 0.15 to 0.55, indicating that the BRET ratio might be affected by the location of tumors, but more importantly, the effect of PD-0325901 was not observed in the TA mutant-expressing tumors, validating the assay for the pharmacodynamics of the MEK inhibitor. To further examine the effect of PD-0325901 at single-cell resolution, we set up *in vivo* FRET imaging under a two-photon excitation microscope (Fig. 5h–j). The FRET ratio from each tumor cell showed a bell-shaped distribution, which was shifted to the left by PD-0325901. The time-course of the decrease in ERK activity was similar to that of the BRET assay, confirming the fidelity of the *in vivo* BRET analysis.

Finally, we generated transgenic mice expressing hyBRET-ERK to examine the pharmacodynamics of the MEK inhibitor in the whole bodies. The transgene was transmitted to offspring by Mendelian inheritance without detectable anomaly. Until the sixth generation of the transgenic mouse lines, the hyBRET biosensor was functional without any detectable change in the expression level. The mouse was anesthetized, set in the spine position, and administered diacetyl coelenterazine-h by continuous intravenous infusion (Fig. 5k). The biosensor was detected ubiquitously. The BRET ratio was higher in the limb than the trunk, probably due to high expression of the biosensor in the muscle. After the injection of PD-0325901, the BRET ratio of whole bodies decreased and reached the minimum in 30 min. Thus, the effect of the MEK inhibitor could be detected non-invasively not only in the implanted tumor tissues but also in the normal tissues of the mice.

## Discussion

Despite the various merits of bioluminescence imaging, most genetically encoded biosensors for intracellular signaling molecules are based on FRET rather than BRET^1,2,4,5,29^. There are three primary reasons for this. First, the bioluminescence signals of BRET biosensors are weaker by at least a few orders of magnitude than the fluorescence signals of FRET biosensors. Second, the bioluminescence requires luciferin, which is often short-lived. Third, optimal designs to achieve a high dynamic range have not been established for BRET biosensors. However, recent advances are alleviating these drawbacks. For example, fusion of a fluorescent protein to RLuc8 or NanoLuc has been shown to improve the quantum efficiency, resulting in increased brightness^13,14,21,22,30^. Moreover, substrates that exhibit prolonged decay times have been developed^6,31,32^ and applied to *in vivo* imaging (Fig. S7)^21^. Finally, by the simple in-frame fusion of RLuc8 into a FRET biosensor, we have herein succeeded in generating a number of BRET biosensors with dynamic ranges comparable to the parental FRET biosensors (Fig. S2), paving the way for the transformation of many FRET biosensors to BRET biosensors. It should be emphasized that a number of FRET biosensors developed to date carry a CFP-derived fluorescent protein at the C-terminus; therefore, such FRET biosensors can be transformed to BRET biosensors by using the Turquoise2-GL-RLuc8 fusion protein as the donor.

How did this simple in-frame fusion of RLuc8 transform the FRET biosensors to BRET biosensors? First, the use of a long flexible linker simplified the mode of action of the FRET biosensors^19^. Here, the distance between the donor and acceptor, but not the orientation of the donor’s and acceptor’s dipole moments, dictates the FRET efficiency^33^. In most FRET and BRET biosensors, the orientation of the donor vs acceptor proteins is not predictable; therefore, eliminating the contribution of the topology by a long flexible linker simplifies the biosensor design. Second, the fluorescent proteins have been extensively refined to achieve the highest FRET efficiency^15,16^. These FRET-optimized fluorescent proteins such as YPet also serve as the dimerizers, which enhance the FRET efficiency^34^, and probably contribute to the increase in BRET in the hyBRET biosensor design as well. Last, in the present biosensor design, the energy transfer from RLuc8 to YFP is almost negligible (Fig. 2), rendering the design of the biosensor easier. So far, we have not succeeded in the development of hyBRET biosensors by using NanoLuc as the donor (Fig. S2). To our surprise, fusion of NanoLuc inhibited the TPA-induced increase in FRET (Fig. S2). Thus, the NanoLuc-CFP fusion proteins may form a stable structure that prevents the biosensor from signal-induced conformational changes.

Optogenetic tools have been extensively used in many research fields^35,36^. Channelrhodopsin-derived optogenetic probes are frequently used in neuroscience, whereas, in cell biology, probes based on light-inducible dimerization by the LOV domain^37^, CRY2^23^, PhyB^38^, or Dropna^39^ are employed to activate the signaling molecules of interest. Currently, however, many optogenetic switches, although not PhyB, respond to blue-to-green light, which hampers the use of FRET biosensors comprising CFP as the donor. By endowing the FRET biosensors with the option of bioluminescence, we can circumvent this technical difficulty and widen the application of the optogenetic tools (Fig. 3). Notably, during the course of these experiments, we discovered a pitfall of the combination of the hyBRET biosensors and CRY2-derived optogenetic tools. Photoactivation of the CRY2-fusion protein at 440 nm wavelength for a long period (~5 s) reversibly decreased luminescent light from RLuc8, ostensibly increasing the BRET ratio. Hoshino *et al*. reported that 76 h illumination of blue light photobleached a fusion protein of YFP and RLuc, BAF-Y^30^. Under this condition, the fluorescence of YFP, but not the bioluminescence of RLuc, was markedly reduced; therefore, this phenomenon differs from our present observations. The absorption peak of coelenterazine-h is 435 nm^40^; therefore, the photon-absorbed coelenterazine-h might induce photochromism of RLuc8, decreasing the bioluminescence of RLuc8. Although we could stimulate the Cry2-fused protein at 490 nm light, it should be kept in mind that the bioluminescence of RLuc could be diminished by blue light.

By the use of diacetyl coelenterazine-h, a coelenterazine-h derivative with a long half-life in the blood, we succeeded in setting up non-invasive *in vivo* measurement of ERK activity of up to 60 min. This assay could potentially be substituted for current methods of determining the pharmacodynamics of drugs. Although the MEK inhibitor decreased the BRET ratio in all tumors and in whole bodies, the BRET ratio was significantly different among the locations of the quantification (Fig. 5). This heterogeneity of the BRET signal may have had any of three causes: (1) heterogeneity of ERK activity in each tumor or tissue, (2) hypoxia in the tumor tissue, which may inhibit the maturation of fluorescent protein, and (3) light absorption and scattering by the tissues^41,42^. Thus, it may be difficult to discuss the difference in the BRET signal among different tumors and organs. Nevertheless, by measuring the difference between before and after drug administration, the BRET imaging provides a versatile *in vivo* measurement of pharmacodynamics.

The transgenic mouse line expressing hyBRET-ERK has not exhibited any detectable anomalies to date. This observation clearly demonstrates that the expression of hyBRET-ERK does not significantly perturb physiological signals. Considering the number of drugs that target the growth factor-Ras-ERK MAP kinase pathway, these transgenic mice would be expected to be useful for the *in vivo* pharmacodynamics. We tested EnduRen™, a coelenterazine derivative that was developed for the long-time BRET measurement^6^. However, the bioluminescence signal by the use of EnduRen was not sufficient for the BRET measurement in our set-up. Thus, in our experimental design, diacetyl coelenterazine-h must be infused continuously over a period of several hours for the pharmacodynamics imaging. Development of a bright and long-lasting derivative of coelenterazine is anticipated. On the other hand, we should make an additional attempt to use the NanoLuc-fused CFP as the donor of hyBRET probes, because furimazine has been used for *in vivo* BRET imaging with a half-life of 40 min^21^.

In conclusion, we have presented a one-step transformation of FRET biosensors to BRET biosensors. The resulting hyBRET biosensors will markedly widen the application of the fleet of FRET biosensors developed by many research groups. Moreover, the hyBRET biosensor-expressing transgenic mice developed in the present study will allow non-invasive visualization of the pharmacodynamics, which will accelerate drug development.

## Materials and Methods

### Plasmid construction

Complementary DNA (cDNA) coding mTurquoise and cDNA coding mTurquoise-GL were obtained from Joachim Goedhart^43^. cDNAs for Turquoise2-GL, mTurquoise2 and Turquoise2 were generated by site-specific mutagenesis by PCR. The following mutations were introduced: I146F (for Turquoise2-GL and mTurquoise2)^43^ and K206A (for Turquoise2-GL). The prototype ERK activity FRET biosensor, EKAREVnes, is comprised of ECFP and YPet for the donor and acceptor fluorescent proteins, respectively^19^. Hereafter, the Turquoise-derived proteins and ECFP are collectively called CFP. Similarly, the YFP-derived proteins including YPet and Venus are collectively called YFP. To construct the hyBRET biosensor for ERK activity, hyBRET-ERK, the cDNA of ECFP in EKAREVnes was replaced with PCR-amplified cDNA coding a fusion protein consisting of Turquoise2-GL dC10, Gly-Thr, and RLuc8 S257G dN3 by using restriction digestion followed by DNA assembly with an In-Fusion HD Cloning Kit (Takara Bio, Otsu, Japan). Similarly, CFPs in PicchuEV-x^19,44,45^ and RaichuEV-Ras^19,46^ were replaced with the tandemly linked Turquoise2-GL dC10 and RLuc8 S257G dN3 to generate hyBRET-PicchuX and hyBRET-HRas, respectively. As a control, we developed EKAREV-4464 by substituting Turquoise2-GL dC10 of EKAREVnes for ECFP. The CFP variants of hyBRET-ERK were generated by replacing Turquoise2-GL dC10 in hyBRET-ERK with mTurquoise2 dC10, Turquoise2 dC10 or ECFP dC10 by In-Fusion reaction. HyBRET-ERK with mVenus-mTurquoise2 pair was created by substituting mVenus dC10 for YPet by restriction fragment ligation. The NanoLuc variants of hyBRET-ERK were also generated by the In-Fusion reaction. CeNL, a fusion protein of Turquoise2 and NanoLuc was reported previously^22^. To develop hyBRET biosensors for other Ser/Thr kinases, the sensor domain and corresponding ligand domain in hyBRET-ERK were replaced with those from AKAR3EV, JNKAR1EV and Eevee-S6K by restriction fragment ligation, generating hyBRET-PKA, JNK, and S6K, respectively. cDNAs encoding the biosensors or the fluorescent proteins were subcloned into pCAGGS vector^47^ or pPBbsr vector, a PiggyBac transposon vector with IRES-bsr (blasticidin S-resistance gene)^48^. A lentiviral vector for hyBRET-ERK was constructed by inserting cDNA coding components of hyBRET-ERK except for YPet into pCSII-EF vector with IRES-bsr (blasticidin S-resistance gene) and codon-optimized YPet for *E*. *coli* to suppress recombination between humanized YFP and CFP^49^. The threonine residue of the ERK substrate in the pCSIIbsr-hyBRET-ERK was then substituted to alanine to create a phosphorylation-resistant mutant, hyBRET-ERK-TA, by restriction digestion and subsequent ligation of the annealed oligo-DNA duplex. To construct vectors for the stable expression of bioluminescent proteins in mammalian cells, cDNA for RLuc8 S257G dN3 was inserted in the CSII-EF vector with IRES-puro and cDNAs for cyan Nano-lantern and yellow Nano-lantern were PCR-amplified and subcloned into pPBbsr vector by restriction fragment ligation. pCX4puro-CRY2-cRaf and pCX4neo-CIBN-EGFPx have been described previously^50^. To generate pCX4puro-mCherry-CRY2-cRaf, cDNA encoding mCherry was amplified by PCR and fused to CRY2 in pCX4puro-CRY2-cRaf by In-Fusion reaction. cRaf in the pCX4puro-mCherry-CRY2-cRaf was then replaced with the linker and catalytic domain of murine Sos1 (a.a. 548–1020) or inter-SH2 domain of human p85 (a.a. 428–621), generating pCX4puro-mCherry-CRY2-Soscat and pCX4puro-mCherry-CRY2-iSH2, respectively. pT2ADW-hyBRET-ERK was constructed as follows: The D4Z4 insulator was inserted before the CAG promoter of pT2AL200R175-CAGGS-EGFP carrying Tol2 recombination sites^51^. Then, the WPRE sequence of pCSII-EF was inserted before the poly A sequence. Finally, EGFP was replaced with the cDNA of hyBRET-ERK.

### Cell culture and establishment of stable cell lines

HeLa cells were purchased from the Human Science Research Resources Bank (Sennanshi, Japan). HCT116 cells and 4T1 cells were obtained from ATCC (American Type Culture Collection). Lenti-X 293T cells were purchased from Clontech (Mountain View, CA). PC9 cells were a kind gift from Masato Okada (Osaka University, Japan). HeLa and Lenti-X 293T cells were maintained in DMEM (Wako Pure Chemical Industries, Osaka, Japan). HCT116 cells were grown in McCoy’s 5A medium (ThermoFisher Scientific, Waltham, MA). 4T1 cells and PC9 cells were cultured in RPMI1640 (ThermoFisher Scientific). The growth media described above were supplemented with 10% heat-inactivated fetal bovine serum (FBS) (Sigma-Aldrich, St. Louis, MO) and penicillin/streptomycin (Nacalai Tesque, Kyoto, Japan). All cells were incubated in a humidified atmosphere of 5% CO2 in air at 37 °C. To establish stable cell lines expressing hyBRET biosensors by a transposon system, cells were co-transfected with pPB vector and pCMV-mPBase^48^, which was obtained from the Wellcome Trust Sanger Institute. One day after transfection, the transfected cells were selected with 20 μg/ml of blasticidin S (InVivoGen, San Diego, CA) and then were further cultured for at least 1 week. For lentiviral production, HEK-293T cells were co-transfected with the pCSII-EF vector, psPAX2, which was obtained from Addgene (plasmid #12260) and pCMV-VSV-G-RSV-Rev, which was a kind gift from Dr. Miyoshi (RIKEN BioResource Center, Ibaraki, Japan) by lipofection using Polyethyleneimine ‘Max’ MW 40,000 (Polyscience Inc., Warrington, PA). Virus-containing media were collected at 48 h after transfection, filtered and concentrated with PEG6000. Target cells were infected in the presence of 10 μg/ml polybrene (Nacalai Tesque). Two days after infection, the infected cells were selected with 20 μg/ml blasticidin S. The bulk population of cells was used in subsequent assays.

### Reagents

Diacetyl coelenterazine-h was synthesized as previously described^14^. Coelenterazine-h and PD-0325901 were obtained from Wako (Osaka, Japan). Gefitinib and AZD6244 were purchased from Symansis (Shanghai, China). Anisomycin, dbcAMP and epidermal growth factor were purchased from Sigma-Aldrich (St. Louis, MO). 293fectin Transfection Reagent was obtained from ThermoFisher Scientific and used for plasmid lipofection into HeLa cells. The Nano-Glo luciferase assay system was purchased from Promega (Madison, WI) and the assay substrate included in the kit was used as a stock solution of furimazine. The luciferins used in each luminescent assay are listed in Table S3.

### Spectroscopy

For the measurement of fluorescence spectra, HeLa cells expressing CFP alone, YFP alone, or a biosensor were plated onto a 35 mm glass-based dish. Cells were observed with an inverted microscope (IX81; Olympus, Tokyo) equipped with an objective lens (UPLAPO 100×/1.35NA oil objective; Olympus). CFP were excited by an FF02-438/24 (Semrock) excitation filter and an FF458-Di02-25x36 (Semrock) dichroic mirror. YFP were excited by an S492/18X (Chroma) excitation filter and a glass dichroic mirror (Olympus). Fluorescence spectra were recorded at 2 nm intervals by using a PMA-12 photonic multichannel analyzer (Hamamatsu Photonics, Hamamatsu, Japan). For the measurement of luminescent spectra, HeLa cells expressing a biosensor were trypsinized and suspended in M199 (ThermoFisher Scientific) containing 3% FBS and 20 mM HEPES. To the cell suspension, 20 μM coelenterazine-h or 3 μM furimazine was added to record luminescence spectra by PMA-12. The obtained fluorescence and luminescence spectra were used for the estimation of energy transfer efficiencies of the biosensors.

### Estimation of energy transfer efficiencies

To estimate energy transfer efficiencies, the fluorescence and bioluminescence spectra of the hyBRET biosensor were fitted with theoretical emission spectra essentially as reported previously^20^. The Lerbenberg-Marquardt method implemented in the nlinfit function in MATLAB (MathWorks, Natick, MA) and solver function in Microsoft Excel (Microsoft Corporation, Redmond, WA) were used for the non-linear regression. The theoretical fluorescence spectra are described by the following equation:

1 $${\rm{F}}(\lambda )={\varepsilon }_{c}({\lambda }_{ex})\{(1-{E}_{CY})\times {\varphi }_{C}\times {f}_{C}(\lambda )+{E}_{CY}\times {\varphi }_{Y}\times {f}_{Y}(\lambda )\}+{\varepsilon }_{Y}({\lambda }_{ex})\times {\varphi }_{Y}\times {f}_{Y}(\lambda ),$$

where F(*λ*) is the fluorescence at wavelength λ, *ε*_*c*_(*λ*_*ex*_) is the excitation coefficient of CFP at the excitation wavelength, *E*_*CY*_ is the FRET efficiency between CFP and YFP, *ϕ*_*C*_ is the quantum efficiency of CFP, *f*_*C*_(*λ*) is the normalized emission of CFP at wavelength λ, *ϕ*_*Y*_ is the quantum efficiency of YFP, *f*_*Y*_(*λ*) is the normalized emission of YFP, and *ε*_*Y*_(*λ*_*ex*_) is the extinction coefficient of YFP at the excitation wavelength. The *ε*_*Y*_(*λ*_*ex*_) value at 440 nm, which represents cross-excitation of YFP in the FRET mode, is 2.9% of the maximum *ε*_*Y*_(*λ*_*ex*_) value at 513 nm.

Assuming that the in-frame fusion of RLuc8 at the C-terminus did not change the physical property of the biosensors, the theoretical bioluminescent spectra are described by the following equation:

2 $$\begin{array}{rcl}{\rm{B}}(\lambda ) & = & {E}_{RC}\times (1-{E}_{CY})\times {\varphi }_{C}\times {f}_{C}(\lambda )+({E}_{RY}+{E}_{RC}\times {E}_{CY})\times {\varphi }_{Y}\times {f}_{Y}(\lambda )\\  &  & +\,(1-{E}_{RC}-{E}_{RY})\times {\varphi }_{R}\times {f}_{R}(\lambda ),\end{array}$$

where B(*λ*) is the bioluminescence intensity at wavelength λ, *E*_*RC*_ is the BRET efficiency between RLuc8 and CFP, *E*_*RY*_ is the BRET efficiency between RLuc8 and YFP, *ϕ*_*R*_ is the quantum efficiency of RLuc8, and *f*_*R*_(*λ*) is the normalized emission of RLuc8.

*E*_*CY*_ was estimated by non-linear fitting of the measured fluorescence spectra to Eq. 1. *E*_*RC*_ is assumed to be constant irrespective of the biosensor conformation, because the linker between RLuc8 and CFP was optimized to maximize the BRET efficiency and to stabilize the structure of the RLuc8-CFP fusion protein. Therefore, *E*_*RC*_ of Nano-lantern is used for the hyBRET biosensor. The quantum efficiency of cyan Nano-lantern, *ϕ*_*CNL*_, is expressed by the following equation:

3 $${\varphi }_{CNL}={\varphi }_{C}\times {E}_{RC}+{\varphi }_{R}\times (1-{E}_{RC}),$$

By using the quantum efficiency data shown in Table S1, *E*_*RC*_ was determined to be 0.13. Finally, *E*_*RY*_ was estimated by fitting of the measured bioluminescent spectra to Eq. 2. The ε and *ϕ* values are listed in Table S1.

Energy transfer rates of NanoLuc-containing biosensors were determined similarly to RLuc8-containing biosensors by non-linear fitting of the measured fluorescence spectra to Eq. 4.

4 $$\begin{array}{rcl}{\rm{B}}(\lambda ) & = & {E}_{NC}\times (1-{E}_{CY})\times {\varphi }_{C}\times {f}_{C}(\lambda )+({E}_{NY}+{E}_{NC}\times {E}_{CY})\times {\varphi }_{Y}\times {f}_{Y}(\lambda )\\  &  & +\,(1-{E}_{NC}-{E}_{NY})\times {\varphi }_{N}\times {f}_{N}(\lambda ),\end{array}$$

where B(*λ*) is the bioluminescence intensity at wavelength λ, *E*_*NC*_ is the BRET efficiency between NanoLuc and CFP, *E*_*NY*_ is the BRET efficiency between NanoLuc and YFP, *ϕ*_*N*_ is the quantum efficiency of NanoLuc, and *f*_*N*_(*λ*) is the normalized emission of NanoLuc. Note that the BRET efficiency between NanoLuc and CFP, *E*_*NC*_, was also estimated by Eq. 4, setting *E*_*NY*_ and *E*_*CY*_ as zero.

### Time-lapse imaging of cultured cells

Time-lapse images were obtained and processed using essentially the same conditions and procedures as previously reported^52^. Briefly, HeLa cells expressing hyBRET biosensors were starved for 3–8 h with FluoroBrite DMEM (Thermo Fischer Scientific, Waltham, MA) supplemented with 0.1% bovine serum albumin (BSA), 1 mM sodium pyruvate (Thermo Fischer Scientific), GlutaMax and penicillin/streptomycin. Starved cells were treated with stimulus during the course of time-lapse imaging if necessary. Cells were imaged with an inverted microscope (IX83; Olympus, Tokyo) equipped with an objective lens (UPlanSApo 60×/1.35NA oil objective; Olympus), an illumination system (Spectra-X light engine; Lumencore, Beaverton, OR), an IX3-ZDC2 laser-based autofocusing system (Olympus), an MD-XY30100T-Meta automatically programmable XY stage (SIGMA KOKI, Tokyo) and an INUG2F-IX3W stage-top incubator (Tokai Hit, Fujinomiya, Japan). One of the following cameras was used in each experiment: MD-695 cooled CCD (Molecular Devices, Sunnyvale, CA), iXon Ultra 888 cooled EMCCD (ANDOR, Belfast, UK), and Rolera Thunder cooled EMCCD (QImaging, Surey, BC). For FRET imaging, cells were exposed to 440 nm light with a light intensity of 25 μW/cm^2^ for 30–200 ms, and FRET and CFP images were obtained. For BRET imaging, 20 μM coelenterazine-h or 3 μM furimazine (Promega) was added to the cultured dish before the start of imaging. Cyan luminescence and yellow luminescence from cells were recorded with exposure times ranging from 6 to 30 s, depending on the camera used in each experiment. To decrease stray light from the microscope and environment during BRET imaging, the hardware blackout mode in IX83 was turned on, the top of the stage heater was covered with aluminum foil and experiments were performed in a dark room. The dichroic mirrors and filters used in this work were an FF458-Di02-25x36 dichroic mirror for CFP and FRET, three emission filters (FF01-483/32-25 for CFP and cyan luminescence, FF01-542/27-25 for FRET, YFP and yellow luminescence and FF01-624/40-25 for mCherry) from Semrock (Rochester, NY) and a U-MREF glass reflector used as a dichroic mirror for YFP and mCherry from Olympus. No dichroic mirror was used for BRET imaging.

### Image Processing

Metamorph software (Molecular Devices) and Safir software (Roper Scientific France, Lisses, France) were used for noise reduction and image analyses. After background subtraction, FRET/CFP ratio images were created and represented in the intensity-modulated display (IMD) mode. In the IMD mode, eight colors from red to blue are used to represent the FRET/CFP ratio, with the intensity of each color indicating the mean intensity of FRET and CFP channels. For luminescent images, background subtraction was preceded by cosmic ray removal and noise reduction^53^. Yellow/cyan luminescence ratio images were then created in the same way as the FRET/CFP ratio images. Dual wavelength images obtained in the whole-body luminescent imaging were split between yellow and cyan luminescence images after the noise removal and background subtraction. In Fig. S7, signals from cyan Nano-lantern and yellow Nano-lantern were separated by linear unmixing.

### Optogenetics

HeLa cells were transfected with an expression vector for a hyBRET biosensor, pCX4neo-CIBN-EGFP-x, and an expression vector for a CRY2-fusion protein. One day after the transfection, cells were starved with FluoroBrite DMEM supplemented with 0.1% bovine serum albumin (BSA), 1 mM sodium pyruvate and GlutaMAX for 3–8 h. Before the start of imaging, 20 μM coelenterazine-h was added to the culture dish. The mCherry channel was used to find focus and stage positions in order not to trigger membrane translocation of the CRY2-fused signaling molecules. BRET imaging was performed as described above. At time-point 0, the cells were illuminated with 490 nm light (55 μW/cm^2^) for 100 ms. To assess the effects of blue light illumination on the BRET ratio of hyBRET sensors and luminescence intensity of RLuc8, cells were transfected with pCAGGS-hyBRET-ERK or pCSIIpuro-RLuc8. The next day, cells were starved and luminescent imaging was performed as described above. During the imaging, cells were illuminated with 440 nm light (200 or 450 μW/cm^2^) or 490 nm light (180 μW/cm^2^) for 5 s at predetermined time-points. The density of light was measured by a TQ8230 optical power meter (Advantest).

### Microplate reader assays

Cells expressing hyBRET-ERK were plated on 96-well white plates at a cell density of 3,000 cells/well. The next day, cells were treated with serially diluted AZD6244, an MEK inhibitor, for 20 min. An HP D300 Digital Dispenser (Tecan, Männedorf, Switzerland) was used for drug dilution and addition to the well. After the drug treatment, media containing 1 μM coelenterazine-h with serially diluted inhibitor were added to each well and yellow luminescence and cyan luminescence were measured by a GloMax Discover microplate reader (Promega). The 495 nm short pass filter was used for cyan luminescence and the 530 nm long filter was used for yellow luminescence. In the multiplexed assay shown in Fig. 4c–f, the medium was exchanged with 250 μl of FluoroBriteDMEM supplemented with 10% FBS, 1 mM sodium pyruvate, GlutaMAX and penicillin/streptomycin after cells attached to the bottom of the well. A few hours after the medium change, cells were treated with serially diluted gefitinib for 1 day. Cyan luminescence and yellow luminescence from drug-treated hyBRET-ERK cells were measured in the presence of 1 μM coelenterazine-h. The yellow luminescence/cyan luminescence ratio was calculated as an index of ERK activity and the sum of yellow luminescence and cyan luminescence was calculated to indicate the number of living cells in each well. To assess the suitability of the assay, the Z’ factor was calculated according to the following equation:

5 $${\rm{Z}}^{\prime} =1-\frac{3(S{D}_{AZD0}+S{D}_{AZD1})}{Av{e}_{AZD0}-Av{e}_{AZD1}}$$

Here, SD is the standard deviation and Ave is the average of yellow/cyan luminescence from 3 wells treated with no AZD6244 (AZD0) or 1 μM of AZD6244 (AZD1). In Fig. 4f, theoretical functions described as shown below were used as model functions to fit the experimental data as reported previously^26^. The equations represent the relationship between ERK activity and the live cell number.

6 $$({\rm{number}}\,{\rm{of}}\,{\rm{live}}\,{\rm{cells}})=mi{n}_{L}+\frac{am{p}_{L}\times IC{50}_{L}^{n{H}_{L}}}{{X}^{n{H}_{L}}+IC{50}_{L}^{n{H}_{L}}}$$

7 $${\rm{X}}={(\frac{am{p}_{ERK}\times IC{50}_{ERK}^{n{H}_{ERK}}}{ERK-mi{n}_{ERK}}-IC{50}_{ERK}^{n{H}_{ERK}})}^{\frac{1}{n{H}_{ERK}}}$$

Here, min is the minimum, amp is the amplitude, IC50 is the half maximal inhibitory concentration and nH is the Hill coefficient of the number of living cells (L) or ERK activity (ERK). ERK is the yellow/cyan luminescence ratio of hyBRET-ERK. Because the minimums and amplitudes were determined unambiguously from experimental data, the IC50s and nHs were fitted as free parameters.

### Xenograft tumor formation and generation of transgenic mice

Female BALB/c nu/nu mice aged 7–9 weeks (Japan SLC, Hamamatsu, Japan) were used for xenograft tumor formation. The 1 × 10^6^ HeLa cells stably expressing yellow Nano-lantern and cyan Nano-lantern in 50 μl GelTrex/PBS (1:1) (ThermoFisher Scientific) were subcutaneously injected on the left and right flanks of the mice. Mice were imaged two weeks after transplantation. For the tumor metastasis study, mice were intravenously injected with 1 × 10^5^ 4T1 murine breast tumor cells stably expressing hyBRET-ERK or hyBRET-ERK-TA in PBS in the tail vein and analyzed 2–3 weeks after the 4T1 tumor injection. Transgenic mice were generated by Tol2-mediated gene transfer^54^. Briefly, fertilized eggs derived from Jcl:B6C3F1 (B57BL/6N Jcl X C3H/HeN Jcl) mice were microinjected with a mixture of Tol2 mRNA and the pT2ADW- hyBRET-ERK. Founder animals were bred with Jcl:ICR mice to produce stable lines. Newborn mice were illuminated with a blue flashlight LEDGFP-3W (Optocode, Tokyo) and inspected for green or red fluorescence through yellow-colored glasses. The animal protocols were reviewed and approved by the Animal Care and Use Committee of Kyoto University Graduate School of Medicine (No. 10584, 14079, 15064, 16038, and 17539) and the methods were carried out in accordance with the relevant guidelines and regulations.

### Whole-body bioluminescent imaging of animals

For the comparison of bioluminescence lifetimes between coelenterazine-h analogs *in vivo* (Fig. S7), mice with subcutaneous tumors were anesthetized with isoflurane (1.5% inhalation, 0.5 L/min) and intravenously administered coelenterazine-h (80 μg per mouse) in ethanol/PBS (1:4) or diacetyl coelenterazine-h (80 μg per mouse)/Pluronic F-127 (20% w/v in DMSO) (Biotium, Hayward, CA) (1:1) dissolved in PBS. For the luminescent imaging of mice with metastasized tumors (Figs 5a–j and S8), the mice were anesthetized with isoflurane (1.5% inhalation, 0.5 L/min) and injected intravenously with a mixture of diacetyl coelenterazine-h (200 μg per mouse) and vehicle or 5.0 mg/kg PD-0325901 in Pluronic F-127 (20% w/v in DMSO)/PBS (1:1) (the total volume was 100 μl/mouse). For the imaging of transgenic mice expressing hyBRET-ERK (Fig. 5k), the transgenic mice were anesthetized with isoflurane (1.5% inhalation, 0.5 L/min) and administered 1 mM diacetyl coelenterazine-h dissolved in PBS containing 1% Pluronic F-127 (20% w/v in DMSO) by continuous intravenous infusion at 90 μL/hr with a Model 11 plus syringe pump (Harvard Apparatus, Holliston, MA). Vehicle or 5.0 mg/kg PD-0325901 (the total volume was 100 μl/mouse) was injected intraperitoneally during imaging. Mice were imaged with an MIIS imager (Molecular Devices) equipped with an iXon Ultra 888 EMCCD camera (ANDOR), a W-VIEW GEMINI image splitting optics (Hamamatsu Photonics), a XT640-W LED illumination system (Lumen Dynamics, Missisauga, ON) and a TEC-55 monofocal telecentric lens (Computar, Cary, NC) controlled by Metamorph software (Molecular Devices). During luminescent imaging, the mice were kept anesthetized with isoflurane and kept warm by a Chamlide pre-heating plate (Live Cell Instrument, Seoul, Korea). Yellow luminescence and cyan luminescence from tumors were recorded under the following conditions: exposure time of 30 s (for subcutaneous tumors) or 4 min (for metastasized tumors), EM gain of 1,000 (maximum) and CCD binning of 1. The dichroic mirror and emission filters mounted in the W-VIEW GEMINI for dual-color *in vivo* luminescent imaging were an FF509-Di01-25x36 dichroic mirror and two emission filters (FF01-483/32-25 for cyan luminescence and FF01-542/27-25 for yellow luminescence) and were obtained from Semrock.

### Intravital FRET imaging of the mouse lung

The 4T1 metastatic tumor-bearing mice were anaesthetized with isoflurane (1% inhalation, 0.5 L/min). A portion of the skin on the left chest was incised to expose the superficial muscle layer and ribs. The throats of the mice were incised along the pharyngeal midline to reveal the tracheal tube, and a 22-G angiocatheter SURFLO (Terumo, Tokyo) was inserted to the tracheal tube. The mice were then placed in the right lateral decubitus position and the left ribs were resected to expose the left lung. The mice were immediately connected to an MK-V100 mechanical ventilator (Muromachi Kikai, Tokyo). Until the end of imaging, breath was provided mechanically under the following conditions: 55 bpm, 35 ml/min, inspiratory/expiratory ratio of 3:2, isoflurane 1.5%. To reduce motion artifacts caused by respiration, the exposed area of the left lung was gently sucked and immobilized on a coverslip by using a custom-made organ stabilizer, which was connected to a vacuum pump. Tumors in the lung were imaged with a FV1200MPE-BX61WI upright microscope (Olympus) equipped with an XLPlanN 25× water-immersion objective lens (Olympus), an InSight DeepSee laser (Spectra-Physics, Santa Clara, CA), and a FV1200MPE Reflected Four Channel GaAsP NDD external detector unit (Olympus). CFP was excited by a 840 nm-laser. The dichroic mirrors used were DM450, DM570 and DM505 (Olympus). The emission filters used were FF01-425/30 (Semrock), BA460–500 (Olympus) and BA520–560 (Olympus) for SHG, CFP and FRET, respectively. During FRET imaging, mice were intravenously injected with 5.0 mgkg^−1^ PD-0325901 without interrupting the imaging.

## Electronic supplementary material

Supplementary information
