# Supplementary material for: A platform of BRET-FRET hybrid biosensors for optogenetics, chemical screening, and in vivo imaging
Source: Sci Rep. 2018 Jun 12;8:8984. doi: 10.1038/s41598-018-27174-x (PMC5997707; doi:10.1038/s41598-018-27174-x)
Supplement: Supplementary file 1 — Supplementary information [file 41598_2018_27174_MOESM1_ESM.pdf]

Supplementary Information:

A platform of BRET-FRET hybrid biosensors for optogenetics, chemical screening, and in vivo imaging

Naoki Komatsu[1,7], Kenta Terai[1], Ayako Imanishi[1], Yuji Kamioka[2,8], Kenta Sumiyama[3], Takashi Jin[4], Yasushi Okada[5], Takeharu Nagai[6] and Michiyuki Matsuda[1,2,*]

1. Laboratory of Bioimaging and Cell Signaling, Graduate School of Biostudies, Kyoto University, Japan
2. Department of Pathology and Biology of Diseases, Graduate School of Medicine, Kyoto University, Japan
3. Laboratory for Mouse Genetic Engineering, Quantitative Biology Center, RIKEN, Osaka, Japan
4. Laboratory for Nano-Bio Probes, Quantitative Biology Center, RIKEN, Osaka, Japan
5. Laboratory for Cell Polarity Regulation, Quantitative Biology Center, RIKEN, Osaka, Japan
6. The Institute of Scientific and Industrial Research, Osaka University, Osaka, Japan
7. Current address: Laboratory for Cell Function Dynamics, Brain Science Institute, RIKEN, Wako-city, Saitama, Japan
8. Current address: Department of Molecular Genetics, Institute of Biomedical Science, Kansai Medical University, Hirakata, Japan

Supplementary Table S1. Quantum efficiency and extinction coefficient of fluorescent or luminescent proteins.

	CFP	YFP	Luminescent proteins			
	Turquoise2 -GL[a]	YPet[b]	RLuc8 S257G[c]	cyan Nano-lantern[d]	Nano Luc[e]	CeNL[e]
ϕ	0.93	0.77	0.020	0.14	0.28	0.42
ε (10^3 $M^{-1}cm^{-1}$)	30	100	-	-	-	-

[a]Goedhart, J. et al. Nature comm. 3, 751 (2012).

[b]Nguyen, A.W. & Daugherty, P.S. Nature biotech. 23, 355-360 (2005).

[c]Saito, K. et al. Nature comm. 3, 1262 (2012).

[d]Takai, A. et al. Proc. Natl. Acad. Sci. USA 112, 4352-4356 (2015).

[e]Suzuki, K. et al., Nature comm. 7, 13718 (2016).

Supplementary Table S2. Energy transfer rates of hyBRET-ERK.

	without TPA	with TPA
E_{CY}[a]	0.29 +/- 0.041	0.44 +/- 0.050
E_{RY}[a]	0.00065 +/- 0.0029	-0.00048 +/- 0.0030
E_{RC}[b]	0.13	0.13

[a]Estimated by non-linear fitting function of MATLAB by using the fluorescence spectra shown in Supplementary Fig. 2b .

[b]Calculated from quantum efficiencies of RLuc8, mTurquoise2, and Cyan-Nano-lantern. Takai, A. et al. Proc. Natl. Acad. Sci. USA 112, 4352-4356 (2015)

Supplementary Table S3. Summary of substrates/administration methods used for luminescence assays.

	Samples	Observation systems	Detectors	Biosensors (Luciferase in sensor)	Expression / transplantation methods	Substrates (concentration)	Administration methods	Figures
Cells	HeLa cells	IX83 inverted microscope	MD-695 CCD / iXon Ultra 888 EMCCD	hyBRET sensors (RLuc8)	Stable / Transient	Coelenterazine-h (20 μM)	Addition to the culture medium	1c-1h,S1,S3,3b-d, 3f-h, S4, S5
	HeLa cells	IX81 inverted microscope	PMA-12 photonic multichannel analyzer	hyBRET-ERK sensors (RLuc8)	Transient	Coelenterazine-h (20 μM)	Addition to the culture medium	2d, S2
	HeLa cells	IX81 inverted microscope	PMA-12 photonic multichannel analyzer	hyBRET-ERK sensors (NanoLuc)	Transient	Furimazine (3 μM)	Addition to the culture medium	2d, S2
	HCT116 cells	Glomax Discover microplate reader		hyBRET-ERK (RLuc8)	Stable	Coelenterazine-h (1 μM)	Addition to the culture medium	4b
	PC9 cells	Glomax Discover microplate reader		hyBRET-ERK (RLuc8)	Stable	Coelenterazine-h (1 μM)	Addition to the culture medium	4d-f, S6
Mice	Balb/c nude mouse	MIIS imager	iXon Ultra 888 EMCCD	Yellow Nano-lantern, cyan Nano-lantern (RLuc8)	Xenografted HeLa cells expressing chemiluminescent probes	Diacetyl coelenterazine-h (80 μg/mouse) in pluronic F-127/PBS(1:1)	Intravenous injection	S7b, S7d

	Balb/c nude mouse	MIIS imager	iXon Ultra 888 EMCCD	Yellow Nano-lantern, cyan Nano-lantern (RLuc8)	Xenografted HeLa cells expressing chemiluminescent probes	Coelenterazine-h (80 µg/mouse) in ethanol/PBS(1:4)	Intravenous injection	S7c, S7e
	Balb/c nude mouse	MIIS imager	iXon Ultra 888 EMCCD	hyBRET-ERK, hyBRET-ERK-TA (RLuc8)	Xenografted 4T1 cells expressing hyBRET-ERK	Diacetyl coelenterazine-h (200 µg/mouse) in pluronic F-127/PBS(1:1)	Intravenous injection	5c-g, S8
	Jcl:B6C3F1 (B57BL/6N Jcl x C3H/HeN Jcl) mouse	MIIS imager	iXon Ultra 888 EMCCD	hyBRET-ERK (RLuc8)	Transgenic mice expressing hyBRET-ERK	Diacetyl coelenterazine-h (1 mM) in PBS containing 1% pluronic F-127	Continuous intravenous infusion (90 ml/hour)	5k

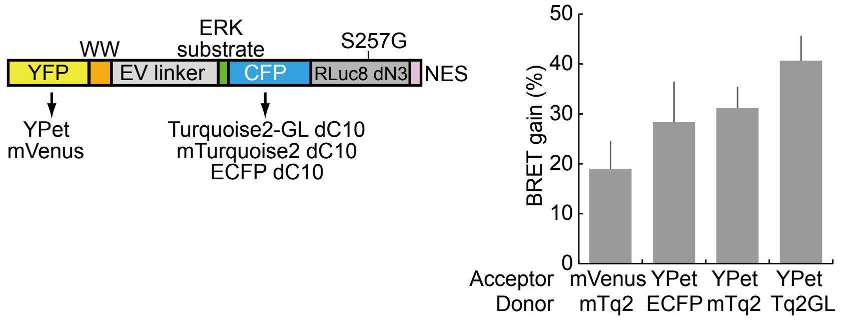

Supplementary Figure S1. Optimization of fluorescent proteins. The structure of hyBRET-ERK is shown. WW, WW domain; NES, nuclear export signal. The EGF-induced increase in the BRET ratio was normalized to the basal BRET ratio and defined as BRET gain. Mean values of at least seven cells are shown with the SD.

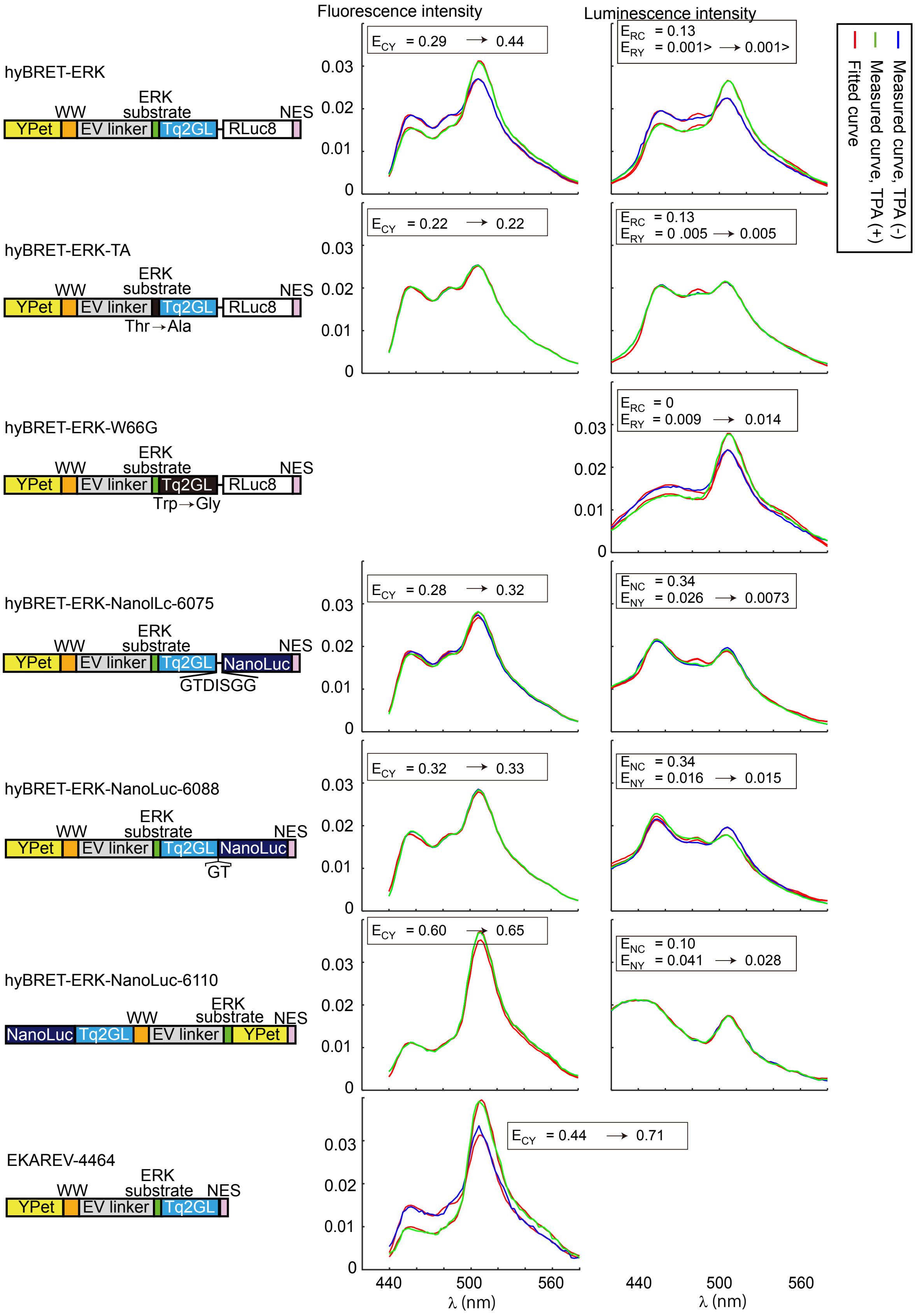

Supplementary Figure S2. Estimation of energy transfer rates. This figure is related to Figure 2. The structure of the prototype hyBRET-ERK and its derivatives are shown on the left. HeLa cells expressing the biosensors were stimulated with 50 nM TPA. Fluorescence and luminescence spectra were recorded by a PMA-12 spectrometer. For the luminescence measurement, 20 μM coelenterazine-h and 3 μM furimazine were used for RLuc8 and NanoLuc, respectively. The spectra were fitted with the spectra of CFP, YFP, RLuc8, and NanoLuc by nlinfit function of MATLAB to estimate the energy transfer rates. Overlays of the measured (blue for pre-TPA and green for post-TPA) and fitted (red) spectra are shown. E_{RC}, E_{RY}, E_{NC}, E_{NY}, and E_{CY}, energy transfer rates from RLuc8 to CFP, RLuc8 to YFP, NanoLuc to CFP, NanoLuc to YFP, and CFP to YFP, respectively. The arrows indicate changes after TPA addition. Note that E_{CY} values in the BRET mode (right) are derived from the ECY values in the FRET mode (left).

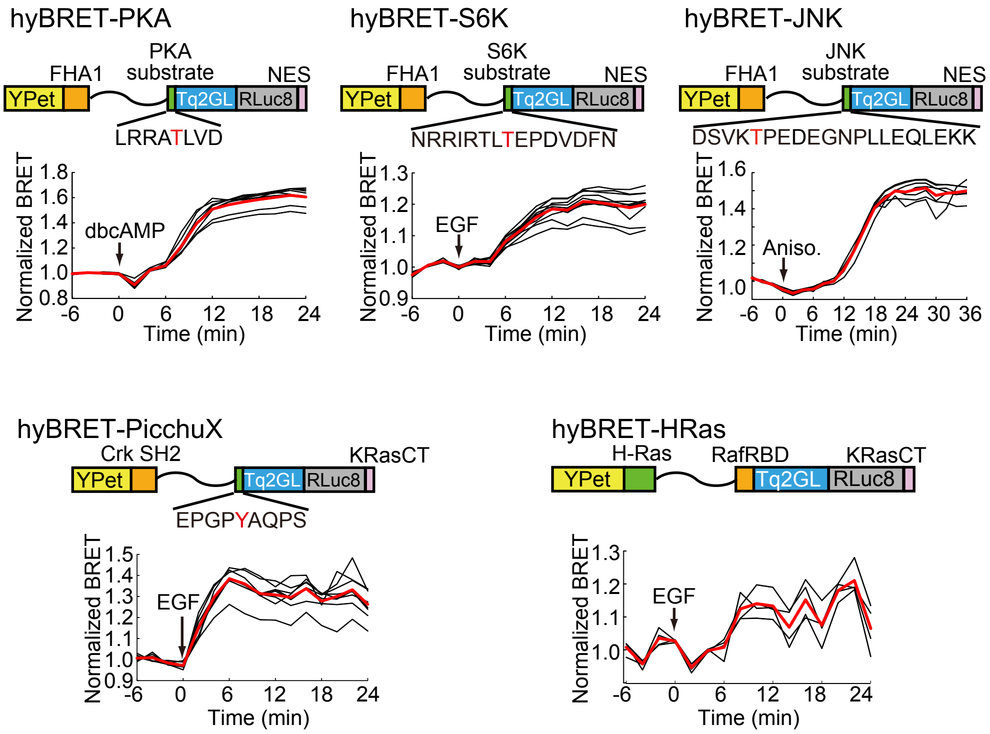

Supplementary Figure S3. Development of hyBRET biosensors for serine threonine kinases, tyrosine kinases, and a small GTPase. HeLa cells expressing hyBRET biosensors were imaged with an inverted microscope equipped with an EM-CCD camera. Coelenterazine-h was added to 20 μM immediately before image acquisition. Stimulants were 1 mM dbcAMP for PKA, 10 ng/ml EGF for S6K, Crk, and HRas, and 1 μg/ml anisomycin for JNK. Representative time-courses of the normalized BRET ratio are shown. Five cells are analyzed for each biosensor. Red lines show the mean value.

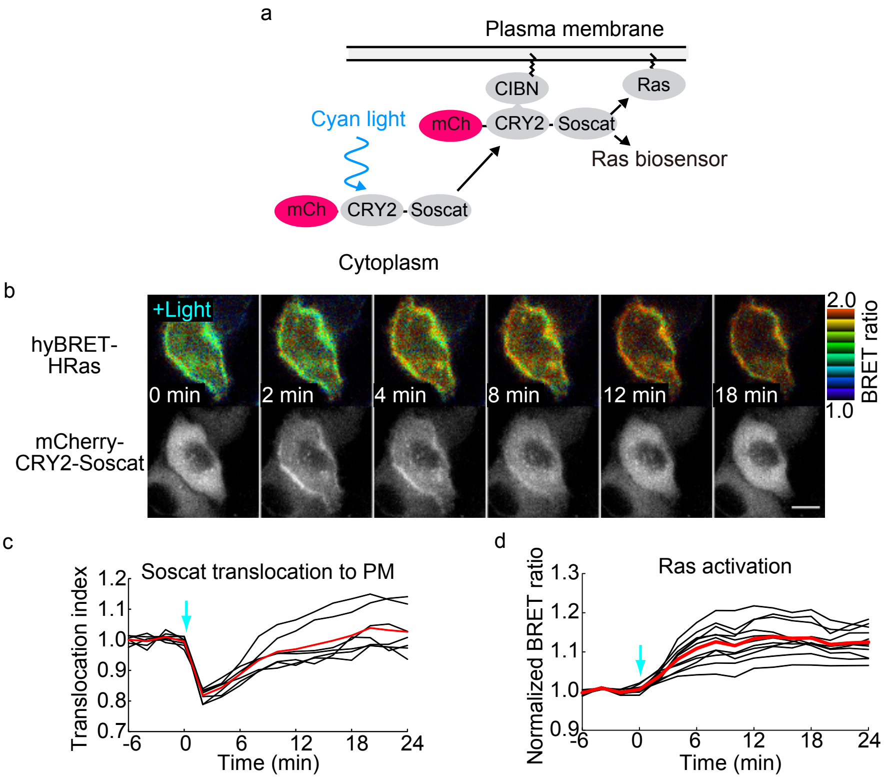

Supplementary Figure S4. Ras activity monitoring during optogenetic activation. (a) Scheme of light-inducible activation and activity monitoring of Ras. (b) Time-lapse images of cells expressing hyBRET-HRas (upper) and mCherry-CRY2-Soscat (lower). Cells were illuminated with 490 nm light at time zero. Bar, 10 μm. Time courses of the cytosolic intensity of mCherry-CRY2-Soscat, which was used as the translocation index (c), and normalized BRET ratio (d) are shown for each cell (black) and for the average of all cells (red).

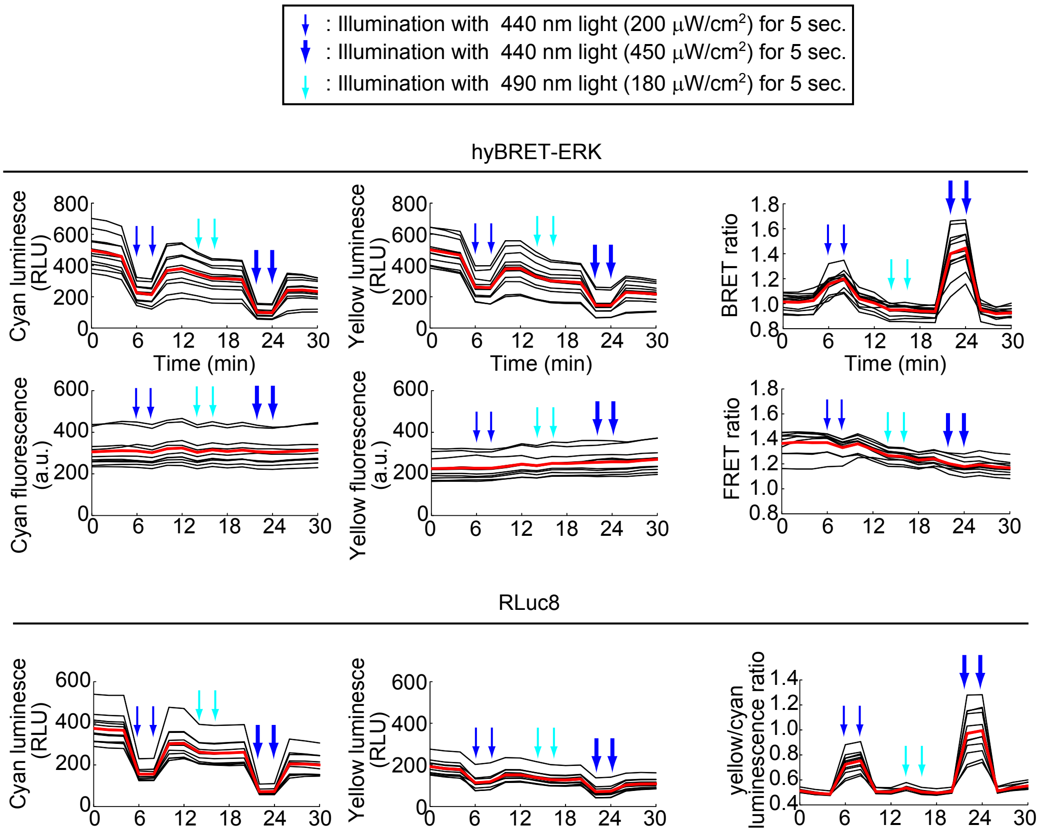

Supplementary Figure S5. Blue light-induced depression of luminescence from RLuc8. HeLa cells expressing hyBRET-EKAR or RLuc8 were imaged for luminescence and fluorescence under an inverted microscope equipped with an EM-CCD camera. Coelenterazine-h was added to 20 μM immediately before image acquisition. For fluorescence imaging, cells were excited at 440 nm for 30 ms. During time-lapse imaging, cells were illuminated at 440 nm or 490 nm for 5 s as indicated by arrows. Time courses of the luminescence intensity, fluorescence intensity, BRET ratio, and FRET ratio are shown with the average (red lines). The yellow/cyan luminescence ratio of RLuc8-expressing cells was obtained with the same filter set as used for the BRET ratio. $n \geq 10$. The conditions used for light illumination are shown in the box.

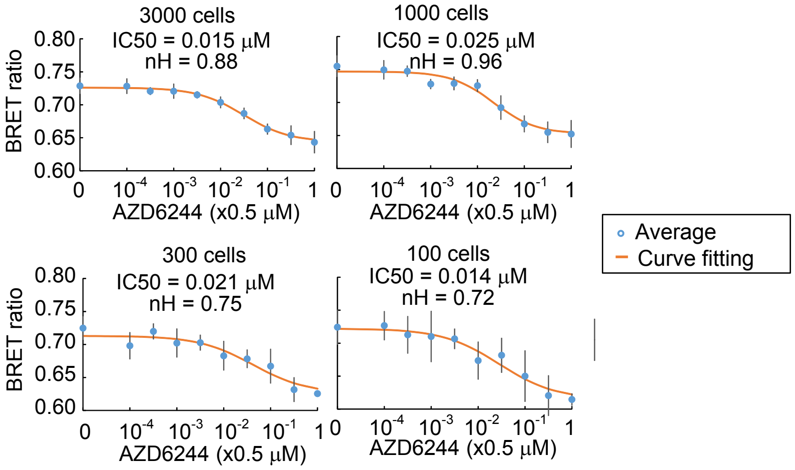

Supplementary Figure S6. Sensitivity of BRET measurement. PC9 cells expressing hyBRET-ERK at the indicated concentration were plated onto a 96-well plate with the parental PC9 cells to maintain 3,000 cells/well. The cells were incubated with serially diluted AZD6244, an MEK inhibitor, for 20 min. After the addition of 1 μM coelenterazine-h, the BRET ratio was measured. The mean BRET ratio values of 8 wells are plotted as dots, and curve fittings using the Hill equation are shown as lines. The IC50 and Hill coefficient values are also shown.

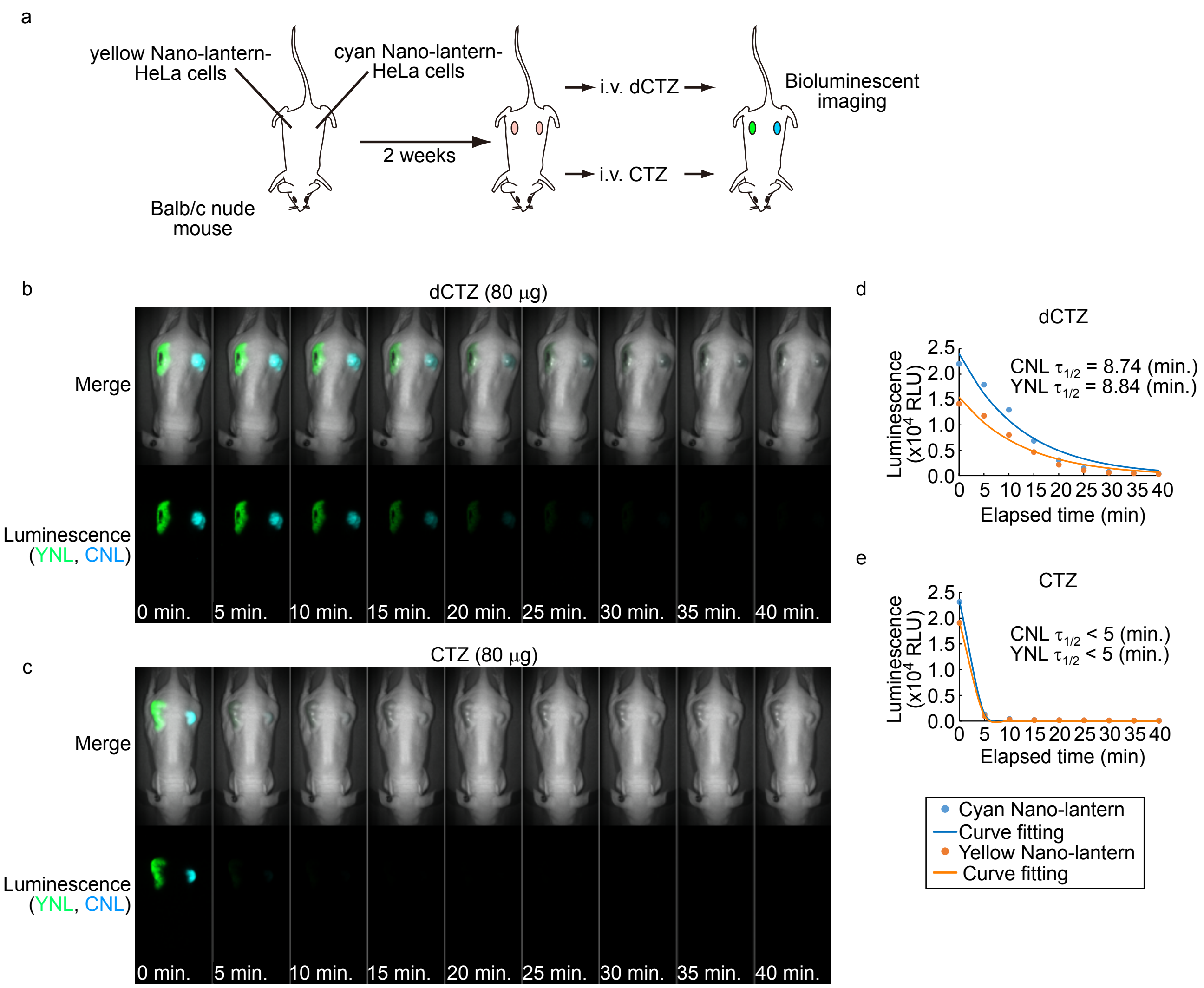

Supplementary Figure S7. In vivo bioluminescence decay curves of diacetyl coelenterazine-h and coelenterazine-h. (a) HeLa cells expressing either yellow Nano-lantern or cyan Nano-lantern were implanted into the subcutaneous tissue of Balb/c nude mice. Two weeks later, the tumors were imaged after the intravenous injection of either diacetyl coelenterazine-h (dCTZ) or coelenterazine-h (CTZ). (b, c) Time-lapse images of tumor-bearing mice injected intravenously with 80 μg of diacetyl coelenterazine-h (b) or coelenterazine-h (c). Overlays of the bright-field image, cyan luminescence image and yellow luminescence image are shown. (d, e) Decay curves of the luminescent intensities of Nano-lanterns.

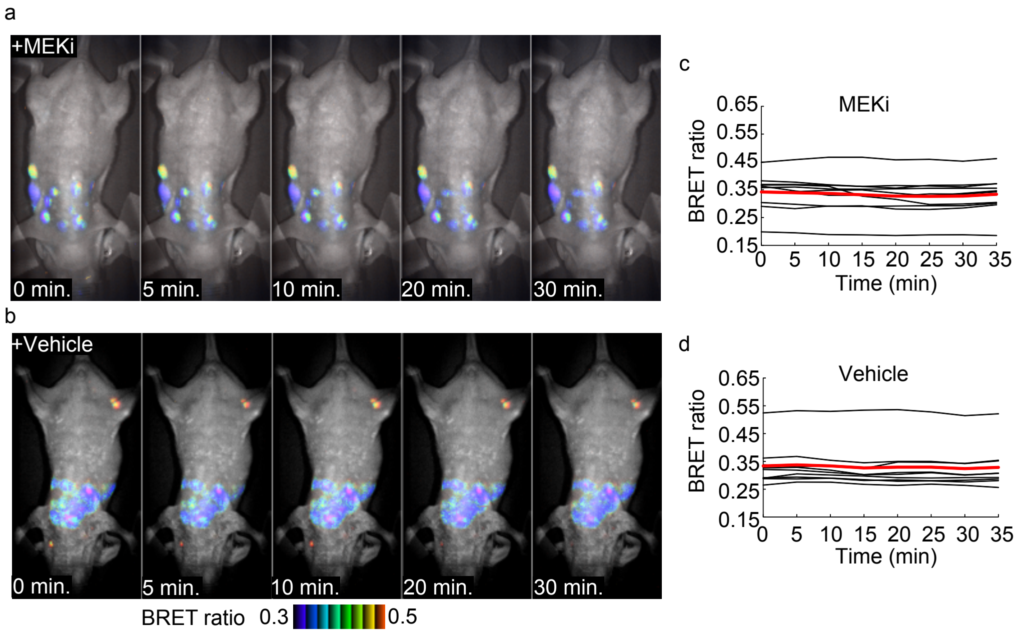

Supplementary Figure S8. Negative controls of in vivo BRET imaging. 4T1 mammary cancer cells expressing hyBRET-ERK-TA, which carries a T-to-A mutation in the substrate region, were intravenously injected into Balb/c nude mice. Before image acquisition, 5 mg/kg diacetyl coelenterazine-h (dCTZ) was injected with or without the MEK inhibitor PD-0325901 (MEKi). (a, b) Time-lapse overlay images of the bright-field image and BRET ratio are shown. (c, d) For each tumor, average BRET ratios are plotted against time with the mean values (red lines).
